# Stillbirth in Canada: anachronistic definition and registration processes impede public health surveillance and clinical care

**DOI:** 10.17269/s41997-021-00483-x

**Published:** 2021-03-19

**Authors:** K.S. Joseph, Lily Lee, Laura Arbour, Nathalie Auger, Elizabeth K. Darling, Jane Evans, Julian Little, Sarah D. McDonald, Aideen Moore, Phil A. Murphy, Joel G. Ray, Heather Scott, Prakesh Shah, Michiel VanDenHof, Michael S. Kramer

**Affiliations:** 1 Department of Obstetrics and Gynaecology, University of British Columbia and the Children’s and Women’s Hospital of British Columbia, 4500 Oak Street, Vancouver, BC V6H 3N1 Canada; 2 Perinatal Services BC, Vancouver, British Columbia Canada; 3 Institut National de Santé Publique du Québec, Université de Montréal, Montréal, Québec Canada; 4 McMaster University, Hamilton, Ontario Canada; 5 University of Manitoba, Winnipeg, Manitoba Canada; 6 University of Ottawa, Ottawa, Ontario Canada; 7 University of Toronto and Sick Kids Hospital, Toronto, Ontario Canada; 8 Perinatal Program of Newfoundland and Labrador, St. John’s, Newfoundland and Labrador Canada; 9 University of Toronto and St. Michael’s Hospital, Toronto, Ontario Canada; 10 Dalhousie University and the IWK Health Centre, Halifax, Nova Scotia Canada; 11 University of Toronto and Mount Sinai Hospital, Toronto, Ontario Canada; 12 McGill University, Montreal, Quebec Canada

**Keywords:** Stillbirth, Fetal death, Definition, Birth registration, Gestational age, Mortinatalité, mort fœtale, définition, enregistrement des naissances, âge gestationnel

## Abstract

The archaic definition and registration processes for stillbirth currently prevalent in Canada impede both clinical care and public health. The situation is fraught because of definitional problems related to the inclusion of induced abortions at ≥20 weeks’ gestation as stillbirths: widespread uptake of prenatal diagnosis and induced abortion for serious congenital anomalies has resulted in an artefactual temporal increase in stillbirth rates in Canada and placed the country in an unfavourable position in international (stillbirth) rankings. Other problems with the Canadian stillbirth definition and registration processes extend to the inclusion of fetal reductions (for multi-fetal pregnancy) as stillbirths, and the use of inconsistent viability criteria for reporting stillbirth. This paper reviews the history of stillbirth registration in Canada, provides a rationale for updating the definition of fetal death and recommends a new definition and improved processes for fetal death registration. The recommendations proposed are intended to serve as a starting point for reformulating issues related to stillbirth, with the hope that building a consensus regarding a definition and registration procedures will facilitate clinical care and public health.

## Résumé

La définition et les méthodes d’enregistrement archaïques des mortinaissances qui prévalent actuellement au Canada entravent à la fois les soins cliniques et la santé publique. La situation est délicate à cause des problèmes de définition que pose l’inclusion des avortements provoqués à ≥ 20 semaines de gestation parmi les mortinaissances : le recours généralisé au diagnostic prénatal et les avortements provoqués en cas d’anomalies congénitales graves ont entraîné une augmentation temporelle artéfactuelle des taux de mortinatalité au Canada et placé le pays dans une position défavorable dans les classements internationaux (de la mortinatalité). Les autres problèmes dans la définition et les méthodes d’enregistrement canadiennes des mortinaissances sont l’inclusion de la réduction fœtale (pour les grossesses multifœtales) parmi les mortinaissances et l’emploi de critères de viabilité inconsistants pour déclarer les mortinaissances. Nous examinons ici l’histoire de l’enregistrement des mortinaissances au Canada, nous justifions une révision possible de la définition de la mort fœtale et nous recommandons une nouvelle définition et des méthodes d’enregistrement améliorées des morts fœtales. Les recommandations proposées se veulent un point de départ à une reformulation des questions liées à la mortinatalité, dans l’espoir que l’établissement d’un consensus sur une définition et sur les méthodes d’enregistrement facilitera les soins cliniques et la santé publique.

## Introduction

Although the global movement to bring stillbirth ‘out of the shadows’ has gained substantial traction (Mullan and Horton 2011), little effort has been made towards rationalizing the definition of stillbirth in Canada. The situation is fraught because of definitional problems related to the inclusion of induced abortions at ≥20 weeks’ gestation as stillbirths: widespread uptake of prenatal diagnosis and induced abortion for serious congenital anomalies has resulted in an artefactual temporal increase in stillbirth rates in Canada and placed the country in an unfavourable position in international (stillbirth) rankings. More importantly, the archaic definition of stillbirth and current registration processes impede both clinical care and public health (Joseph et al. 2015; Joseph et al. 2018). We discuss why Canada’s stillbirth definition should be revised, and recommend a new definition and processes for fetal death registration.

## History

During most of the twentieth century, Canadian vital records defined stillbirth as “the birth of a fetus, after at least 28 weeks’ pregnancy, which, after complete separation from the mother, does not show any sign of life” (Statistics Canada 2020). Gestational age was based on menstrual dating, with that information typically provided by parents. In 1959, the Canadian stillbirth definition was aligned with the World Health Organization’s (WHO) definition of ‘fetal death’, namely, “death prior to the complete expulsion or extraction from its mother of a product of conception, irrespective of the duration of pregnancy; the death is indicated by the fact that after such separation the fetus does not breathe or show any other evidence of life, such as beating of the heart, pulsation of the umbilical cord, or definite movement of voluntary muscles” (WHO 2004). Although modeled on the WHO’s definition of fetal death, the Canadian stillbirth definition included a (new) gestational age cut-off of ≥20 weeks (Statistics Canada 2020).

## Current definition

With the exception of Quebec, all provinces/territories in Canada currently define stillbirth as the birth of a fetus at ≥20 weeks’ gestation or with a birth weight ≥500 g, which shows no signs of life at birth (Statistics Canada 2020). The same definition is used in Quebec, except the viability cut-off is based solely on a birth weight ≥500 g. These definitions differ from those used elsewhere (Joseph et al. 2015), e.g., Australia (≥20 weeks’ gestation or ≥400 g birth weight) and the United Kingdom (≥ 24 weeks’ gestation).

## Temporal trends and regional variation

**Fig. 1:**
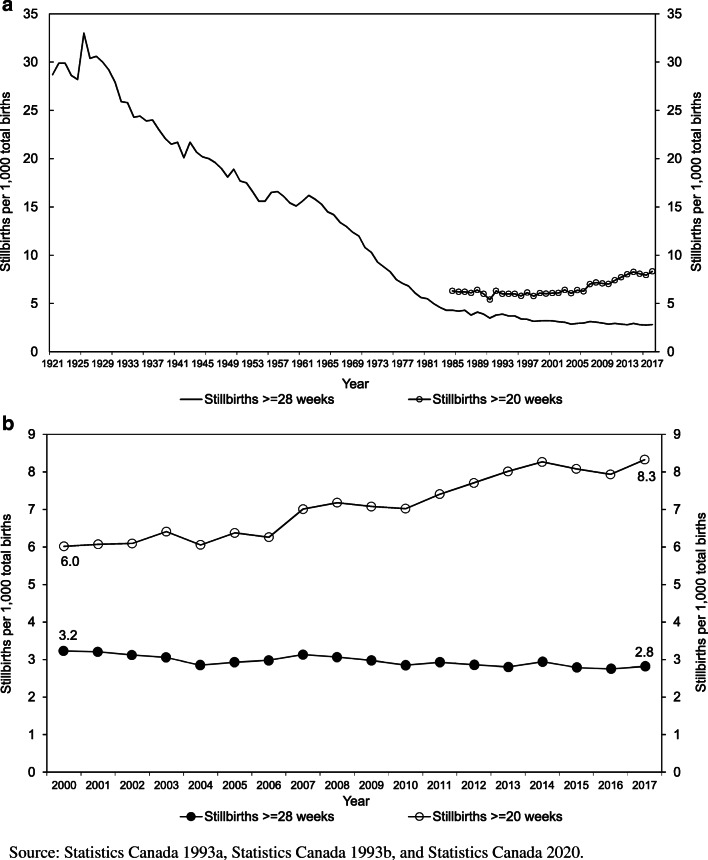
Temporal trends in stillbirth rates ≥28 weeks’ gestation and ≥20 weeks’ gestation in Canada, 1921–2017 (panel **a**) and from 2000 to 2017 (panel **b**)

Rates of stillbirth ≥28 weeks’ gestation ranged between 30 and 35 per 1000 total births in the 1920s and declined steadily to approximately 5 in the mid-1980s and to 2.8 per 1000 total births in 2017 (Fig. 1). In contrast, rates of stillbirth ≥20 weeks’ gestation were stable for several years from 1985 onward and then increased more recently (Fig. 1a, b). Stillbirth rates in 2015–2017 were significantly higher than rates in 2000–2002 in the more populous provinces except Quebec (Table 1), with definitional differences likely explaining the pattern in the latter province (see below).

**Table 1:** Temporal changes in stillbirth rates by province and territory, Canada, 2000–2002 and 2015–2017

Province/territory	2000–2002			2015–2017			2015–2017 vs 2000–2002		
	SB	LB	Rate	SB	LB	Rate	Rate ratio	95% confidence interval	
Newfoundland and Labrador	75	14,236	5.24	83	13,003	6.34	1.21	0.89	1.65
Prince Edward Island	25	4149	5.99	29	4087	7.05	1.18	0.69	2.00
Nova Scotia	188	26,688	7.00	194	24,750	7.78	1.11	0.91	1.36
New Brunswick	120	21,588	5.53	132	19,768	6.63	1.20	0.94	1.54
Quebec†	917	218,179	4.19	1099	257,081	4.26	1.02	0.93	1.11
Ontario	2477	387,645	6.35	4203	419,923	9.91	1.56*	1.49	1.64
Manitoba	354	41,980	8.36	424	50,629	8.31	0.99	0.86	1.14
Saskatchewan	248	36,176	6.81	330	46,285	7.08	1.04	0.88	1.23
Alberta	732	113,316	6.42	1216	166,582	7.25	1.13*	1.03	1.24
British Columbia	862	121,312	7.06	1558	134,200	11.5	1.63*	1.50	1.77
Yukon	8	1053	7.54	14	874	15.8	2.09	0.88	4.96
Northwest Territories	17	1921	8.77	17	1939	8.69	0.99	0.51	1.93
Nunavut	23	2163	10.5	38	2664	14.1	1.34	0.80	2.24
Canada	6047	990,428	6.07	9337	1,141,785	8.11	1.34*	1.29	1.38

*SB*, stillbirths; *LB*, live births

^*^Stillbirth rate in 2015–2017 significantly higher than stillbirth rate in 2000–2002

^†^All provinces and territories in Canada, except Quebec, currently define stillbirth as the birth of a fetus at ≥20 weeks’ gestation or with a birth weight ≥500 g, which shows no signs of life at birth (Statistics Canada 2020). In Quebec, the cut-off for stillbirth is based on a single viability criterion: a birth weight ≥500 g (Statistics Canada 2020)

Data source: Computing in the Humanities and Social Sciences (CANSIM 2018). University of Toronto. Statistics Canada. Ottawa. 2018

## Prenatal diagnosis

**Fig. 2:**
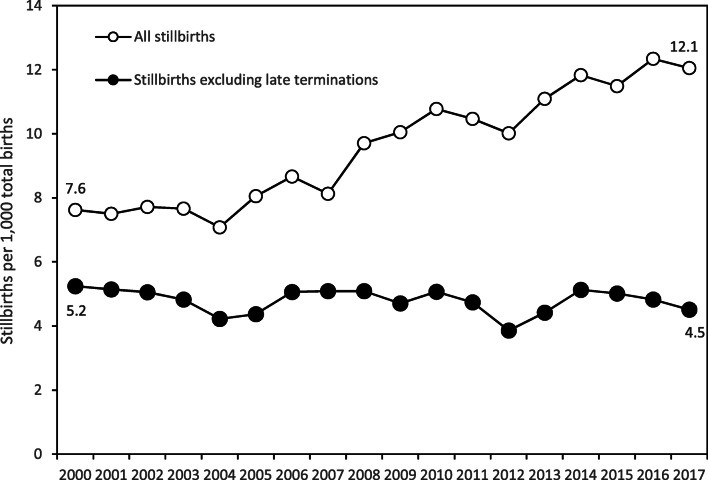
Overall stillbirth rates and stillbirth rates excluding stillbirths due to late induced abortions, British Columbia, Canada, 2000–2017

The widespread uptake of prenatal diagnosis and induced abortion for serious congenital anomalies in Canada led to a substantial change in stillbirth and infant mortality patterns since the 1990s. Stillbirths that followed late induced abortion for serious congenital anomalies at 20–24 weeks’ gestation increased significantly, while spontaneous late stillbirths and infant deaths due to serious congenital anomalies decreased (Liu et al. 2002): the effect was a steady temporal increase in overall stillbirth rates from 2000 to 2017. A study based on British Columbia’s Perinatal Data Registry showed that excluding late induced abortions from stillbirth statistics reversed the rising temporal pattern in overall stillbirth rates (Joseph et al. 2013; Fig. 2). The inclusion of induced abortions following prenatal diagnosis of congenital anomalies has artefactually inflated stillbirth rates in Canada and placed the country in an unfavourable position relative to countries such as Sweden and the United States, which do not include such deaths in stillbirth counts (Joseph et al. 2015).

Another notable feature of the temporal changes in stillbirth rates in British Columbia was the increase in stillbirths with missing birth weight from 1.7 in 2000 to 4.5 per 1000 total births in 2010 (Joseph et al. 2013). This likely reflects an increase in late induced abortions (at 20–24 weeks’ gestation) using a dilation and evacuation procedure, which precludes separate weighing of the fetus and placenta and results in a missing birth weight. Such late induced abortions would have been categorized as stillbirths in British Columbia (per the gestational age viability criterion) but potentially excluded from stillbirth counts in Quebec (due to missing birth weight). This may explain the lack of a temporal increase in stillbirths in Quebec (Auger and Dennis 2012).

## Fetal reduction

The outdated nature of the current stillbirth definition is perhaps best exemplified by fetal reduction procedures sometimes carried out in multi-fetal pregnancy (e.g., triplet pregnancies reduced to twins/singletons). Such fetal reductions are generally carried out at 10–12 weeks’ gestation and significantly reduce perinatal morbidity and mortality (Zipori et al. 2017). However, when the surviving fetus(es) deliver(s) at ≥20 weeks’ gestation, stillbirth registration of the reduced fetus is required at the time of delivery (in addition to registration of the surviving fetus). This is because the gestational age at birth of the reduced fetus would be ≥20 weeks (even if fetal remains cannot be identified).

Requiring parents to complete the paperwork for stillbirth registration, and making burial arrangement for the reduced fetus after the mother has delivered the surviving fetus(es), can be distressing. Not infrequently, parents leave hospital without completing the required paperwork, and nursing staff, who face deadlines for submitting patient-completed stillbirth registration forms to the Office of Vital Statistics, find that their repeated telephone calls further traumatize grieving parents. The Office of Vital Statistics is sometimes left with an incomplete stillbirth registration, and the hospital with abandoned fetal remains. In 2013–2015, 227 pregnancies continued past 20 weeks’ gestation after selective fetal reduction in Canada (excluding Quebec).

## Early fetal loss in multi-fetal pregnancy

Spontaneous demise of a fetus in a multi-fetal gestation, with survival of the other fetus(es), is an increasingly recognized phenomenon detected with routine ultrasound imaging in early pregnancy (Márton et al. 2016). Defining a stillbirth based on gestational age at birth requires registering spontaneously reduced fetuses from multi-fetal pregnancies if the surviving fetus delivers at ≥20 weeks’ gestation. Vanishing twins are, therefore, another reason for changing the surveillance focus from stillbirths at ≥20 weeks’ gestation at birth to fetal deaths at ≥20 weeks’ gestation (i.e., from the gestational age at which the dead fetus was born to the gestational age at which the fetus died).

## Dual birth weight and gestational age criteria

The WHO recommends a single viability criterion for national reporting: birth weight ≥500 g (WHO 2004). This criterion is supplemented with a ≥22 weeks’ gestational age criterion to be used if information on birth weight is missing. In contrast, all provinces and territories in Canada (except Quebec) use dual viability criteria for defining stillbirth (birth weight ≥500 g or gestational age ≥20 weeks). Such criteria are conceptually problematic: a gestational age ≥20 weeks’ gestation is not congruent with a birth weight of ≥500 g, since the median birth weight at 20 weeks’ gestation is 400 g (Kramer et al. 2001). The ≥20 weeks’ gestational age criterion excludes fetuses born at <20 weeks, while the birth weight cut-off (≥500 g) means that variable proportions of fetuses at 18 and 19 weeks’ gestation will be registered.

Similar problems affect the WHO’s and Quebec’s birth weight criterion of ≥500 g: some fetuses at the higher centiles of birth weight-for-gestational age at 18–19 weeks will qualify as stillbirths, while smaller fetuses at 20–24 weeks will not. Focusing on birth weight as a viability criterion is outdated since it is a heterogeneous entity influenced by gestational duration and fetal growth (Kramer 1998). It is increasingly accepted that the viability criterion for stillbirth should be based on gestational duration alone (Blencowe et al. 2016): a ≥20 weeks’ gestation cut-off for fetal death registration would be preferable given reports of neonatal survival at 21 and 22 weeks’ gestation.

## Stillbirth vs fetal death surveillance

Another outdated aspect of the 1950 WHO definition of fetal death is the requirement that birth (i.e., stillbirth) occur before fetal death can be inferred (WHO 2004). Advances in imaging techniques mean that cessation of fetal cardiac activity and other signs of life can be established in utero with virtual certainty (Platt et al. 1980). The age at fetal death has greater etiologic and prognostic significance than the timing of the stillbirth, whereas both may be relevant from a maternal care standpoint. Gestational age at fetal death and gestational age at stillbirth may be separated by hours, days, weeks and occasionally months, and attempts to document the timing of both are important from a clinical care and public health standpoint.

## Registration models

Current stillbirth registration processes in most provinces/territories are based on the live birth registration model, rather than the (child or adult) death registration model. The onus for stillbirth registration, therefore, falls on the parents rather than the health care provider. Modeling fetal death registration on death registration processes would transfer the burden from grieving parents to health care providers, and likely improve the accuracy of the information collected for surveillance purposes. This does not imply that parents have to be excluded from this or other stillbirth-related grieving activities.

## International standards

Variation in reporting criteria bias international comparisons of stillbirth rates (Joseph et al. 2012). Problems with comparing stillbirth rates in high-income countries occur primarily due to differences in viability criteria and the inclusion/exclusion of late induced abortions as stillbirths. Under-ascertainment of stillbirths that occur at gestational ages <28 weeks also plagues surveillance, especially in some countries. For these reasons, international comparisons of stillbirth are often restricted to stillbirths at ≥28 weeks’ gestation (Joseph et al. 2012; Blencowe et al. 2016). However, early stillbirths constitute a substantial and clinically important fraction of spontaneous stillbirths, and their exclusion makes international comparisons less meaningful. Setting a lower viability threshold (e.g., ≥20 weeks’ gestation) for complete ascertainment and reporting of all spontaneous fetal deaths in Canada would permit comparisons with countries that favour such a threshold, and also countries that report fetal death using a higher viability threshold.

## Rationalizing the definition and registration

The recommendations listed below are intended to serve as a starting point for re-thinking the Canadian definition and registration practices related to stillbirth. Although each Canadian province/territory has the freedom to formulate its own definition and processes, common standards would facilitate regional comparisons and benchmarking for quality improvement.

## Recommendations

1. Fetal death should be defined as “death prior to the complete expulsion or extraction from its mother of a post-embryonic product of conception, irrespective of the duration of pregnancy; death in utero may be ascertained by imaging techniques (i.e., visualization of the fetal heart with absence of cardiac activity), or by other evidence of death, such as the absence of vital signs at birth.”
2. Vital registration and public health surveillance should shift its focus from surveillance of stillbirth (i.e., birth of an expired fetus ≥20 weeks’ gestation) to surveillance of fetal death (i.e., death of the fetus at ≥20 weeks’ gestation).
3. All fetal deaths that occur at ≥20 weeks’ gestation should require registration as vital events, or reporting as medically notifiable events (see below).
4. The responsibility for registering a fetal death at ≥20 weeks’ gestation should lie with the attending health care provider. Parents who wish to be involved in the process of registration or with burial and other arrangements should be respected and accommodated.
5. Both the gestational age at fetal death (i.e., age when fetal death occurred) and the gestational age at stillbirth (i.e., age when the expired fetus was delivered) should be documented for fetal deaths at ≥20 weeks’ gestation. In cases where the gestational age at fetal death is unknown, the gestational age at stillbirth should be used as the gestational age at fetal death for documentation purposes.
6. Gestational age should be based on the best obstetric estimate documented by the health care provider and expressed in completed weeks and days.
7. The distinction between a spontaneous fetal death at ≥20 weeks’ gestation and a fetal death due to induced abortion at ≥20 weeks’ gestation should be recognized. The registration of spontaneous fetal deaths and fetal deaths due to late induced abortion should be through independent mechanisms.
8. All spontaneous fetal deaths at ≥20 weeks’ gestation (or ≥400 g birth weight if gestational age is unknown) should be registered as vital events.
9. Induced abortions at ≥20 weeks’ gestation (or ≥400 g birth weight if gestational age is unknown) should be reported provincially, and federally, but not under the vital registration system. They may be considered notifiable medical events (as is the case for communicable diseases such as congenital rubella syndrome).

## Data Availability

Not applicable.
